# Clinical expert guidelines for the management of cough in lung cancer: report of a UK task group on cough

**DOI:** 10.1186/1745-9974-6-9

**Published:** 2010-10-06

**Authors:** Alex Molassiotis, Jaclyn A Smith, Mike I Bennett, Fiona Blackhall, David Taylor, Burhan Zavery, Amelie Harle, Richard Booton, Elaine M Rankin, Mari Lloyd-Williams, Alyn H Morice

**Affiliations:** 1 School of Nursing, University of Manchester, UK; 2 School of Translational Medicine, University of Manchester, UK; 3 School of Health & Medicine, Lancaster University, UK; 4 Department of Medical Oncology, Christie Hospital NHS Trust, Manchester, UK; 5 Department of Thoracic Medicine, Wycombe Hospital, Buckinghamshire, UK; 6 Oncology Pharmacy, Clatterbridge Centre for Oncology NHS Foundation Trust, Bebington, UK; 7 Department of Respiratory Medicine, Wythenshawe Hospital, Manchester, UK; 8 Department of Cancer Medicine, Ninewells Hospital, Dundee, UK; 9 School of Population, Community and Behavioural sciences, University of Liverpool, UK; 10 Department of Academic Medicine (Chest), University of Hull, UK

## Abstract

**Background:**

Cough is a common and distressing symptom in lung cancer patients. The clinical management of cough in lung cancer patients is suboptimal with limited high quality research evidence available. The aim of the present paper is to present a clinical guideline developed in the UK through scrutiny of the literature and expert opinion, in order to aid decision making in clinicians and highlight good practice.

**Methods:**

Two systematic reviews, one focusing on the management of cough in respiratory illness and one Cochrane review specifically on cancer, were conducted. Also, data from reviews, phase II trials and case studies were synthesized. A panel of experts in the field was also convened in an expert consensus meeting to make sense of the data and make clinical propositions.

**Results:**

A pyramid of cough management was developed, starting with the treatment of reversible causes of cough/specific pathology. Initial cough management should focus on peripherally acting and intermittent treatment; more resistant symptoms require the addition of (or replacement by) centrally acting and continuous treatment. The pyramid for the symptomatic management starts from the simpler and most practical regimens (demulcents, simple linctus) to weak opioids to morphine and methadone before considering less well-researched and experimental approaches.

**Conclusion:**

The clinical guidelines presented aim to provide a sensible clinical approach to the management of cough in lung cancer. High quality research in this field is urgently required to provide more evidence-based recommendations.

## 1. Introduction

Cough is a common symptom in about 23-37% of general cancer patients and 47-86% of lung cancer patients [1]. The first author's data on 100 cancer patients assessed using the Memorial Symptom Assessment Scale from the beginning of cancer treatment to 3, 6 and 12 months showed a prevalence of 42.9%, 39.2%, 35.1% and 36.1% respectively, similarly to the experience of breathlessness, although less distressing than breathlessness [2]; these numbers almost doubled in the lung cancer subgroup analysis. Despite such high prevalence, the management of cough remains suboptimal, with little high quality evidence to guide practice. Much of the current practice on the symptomatic management of cough in lung cancer is experiential and primarily is geared around the use of oral opioids. Current guidelines on the management of cough are often broad and non-specific (suggesting difficulty in making any specific recommendations) and either focus on non-cancer respiratory illnesses with different pathophysiology from cancer-related cough, or provide broad reviews of generally poor quality studies [3-7]. Professional societies that have developed guidelines (non-cancer) include the American College of Chest Physicians (ACCP) [3,8], the European Respiratory Society (ERS) [9] and the British Thoracic Society (BTS) [7].

### Rationale for the guidelines

With currently available information, clinicians have difficulty in making appropriate treatment choices, often treating patients on a trial-and-error basis. This has been highlighted through discussions with many clinicians. Our own work with lung cancer patient interviews over time [10] has shown the distressing nature of cough and its significant impact on patients' quality of life as well as the difficulty in obtaining relief from offered treatments. Our consultation with patient research forums has also highlighted the management of cough as an unmet need in lung cancer. For these reasons, the development of clinical guidelines for the management of cough in lung cancer was deemed necessary and timely.

## 2. Process of guidelines development

### 2.1. Formation of the Task Group

The Task Group was led by the Cancer Experiences Research Collaborative (CECo) with invited experts from the Association for Palliative Medicine (APM). CECo (see http://www.ceco.org.uk) is a collaborative of five UK universities and their clinical partners, funded in 2005 by the National Cancer Research Institute to develop critical mass, capacity and high quality research in supportive and palliative care. Besides its research objectives, the Collaborative also has a remit to have the maximum positive impact on policy and practice. The APM (see http://www.palliative-medicine.org) is a professional society aiming to provide a national and regional network for palliative medicine doctors with a strong focus on developing and implementing educational material, and has strong links with the European Association for Palliative Care.

The Task Group consisted of experts that represented palliative medicine, medical/clinical oncology with focus in lung cancer, respiratory medicine, nursing, pharmacy, and supportive care. Experts had clinical, academic and research roles and three members of the group were also running cough clinics in the UK. Members of the group have also significant research publications in relation to cough, considered key experts in this (limited) field. The group met twice, once to agree on the methodology to be used and review the available evidence and once to make sense of this data and develop recommendations.

### 2.2. Evidence searching and selection

A literature review preceded the meetings over the previous year, with two systematic reviews being undertaken, one with a focus on cough and respiratory illnesses (other than cancer) [11] and a second Cochrane review with a focus specifically in cancer [12]. Other publications, including reviews, case study reports and phase II trials that were not part of the systematic reviews were also retrieved and summarized. The group members reviewed the evidence and made recommendations that reflected the evidence found combined with clinical experience in an attempt to provide a sensible approach to the clinician and guide practice in a field with minimal evidence.

### 2.3. Clinical consultation of the guidelines

**Figure 1:**
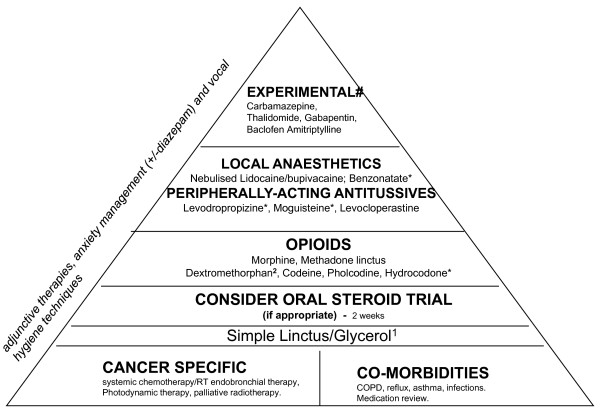
**Treatment pyramid for the management of cough in patients with lung cancer.**^1^(e.g. Benylin tickly coughs; Lemsip cough dry). ^2^(e.g. Actifed dry coughs; Meltus dry coughs; Benylin cough & congestion; Benylin dry coughs; Day & Night Nurse-also includes pholcodine-; Night Nurse; Vicks cold & flu care medinite complete syrup; Robitussin for dry coughs oral or soft pastilles). Dextromethorphan is in variable concentrations in each of these preparations, containing 6.5-11.5 mg/ml. *Not available in the UK and some other countries. #Not recommended, but to consider if everything else has failed.

An assessment of the appropriateness, usability and clarity of the guidelines was carried out by international experts (N = 15), including 9 consultants in palliative medicine, 4 in medical oncology and 2 specialist palliative care nurses (independent prescribers). This was done through clinicians reading the report and recommendations, and completing a feedback form. A number of items structured in a Likert-type format of 1 = strongly disagree to 5 = strongly agree were included. The areas explored were: the rationale and need for the guidelines (original score = 4.6); the need for the development of the guidelines (= 4.8); completeness of literature search (= 4.3); the description of evidence (= 4.6); the methods used to summarise and interpret the evidence (= 4.1); interpretation of the evidence presented (= 4); clarity of the recommendations (= 3.7); agreement with the recommendations made (= 4.1), and feeling comfortable having these guidelines applied in their hospital (= 4). The likelihood of using these guidelines in the reviewers' own practice was explored with a single-item scale ranging from 1 = 'not at all likely to use' to 10 = 'very likely to use', achieving a score of 8.1. After the feedback, the guidelines were modified primarily in relation to the dosages in some drugs, and the clarity and levels of drugs proposed in the cough pyramid (Figure 1).

## 3. Review of the evidence

### 3.1. Pathophysiology and causes of cough

In healthy individuals coughing serves to protect the airway from chemical irritants and foreign bodies. These stimuli provoke coughing by stimulation of afferent C fibres (chemoreceptors) and Aδ fibres (mechanoreceptors) in the airways, carried by the vagus nerve. In disease states, excessive coughing can occur by excessive noxious stimulation of these afferent fibres and/or as a result of sensitization of neurons involved in the cough reflex. In patients with lung cancer, for example, tumour tissue in the central airways may cause mechanoreceptor stimulation directly or indirectly via obstruction and sputum accumulation. The inflammatory mediators associated with infection distal to such an obstruction or mediators released by tumour tissue may further induce coughing by sensitizing peripheral nerves.

Patients with lung cancer can experience cough as a result of non-cancer related underlying pathology or cancer itself. The cancer-related causes of cough can include a direct effect of the tumour mass (ie. obstruction), pleural or pericardial effusion, atelectasis, infections, oesophagorespiratory fistulas, lympangitic carcinomatosis, superior vena cava syndrome and treatment-induced cough as a result of radiotherapy or more rarely chemotherapy [5].

### 3.2. Quality and relevance of identified clinical studies

Positive and negative trials from our systematic review of cough management in respiratory and non-respiratory diseases are summarized in Table 1, summarising data from 75 trials. The key points from this review were that several pharmacological approaches and one non-pharmacological (speech pathology training) had the potential of improving the experience of cough [11]. However, as only 20/75 trials had cough as a primary outcome being primarily trials focusing on a respiratory pathology, the reliability of the outcome measurement was debatable in the vast majority of trials, and a significant proportion of trials had methodological and quality problems.

**Table 1:** Summary of evidence

	Disease group & number of trials	Interventions with positive data	Interventions with negative data
Overview of findings from systematic review in respiratory diseases [11]*	1. Asthma (cough often secondary outcome) N = 23 trials, 1508 subjects	Steroids (particularly Beclomethasone-4 trials-and Budesonide-1 trial) Disodium Cromoglycate-1 trialLodoxadine-1 trial Nedocromil sodium-2 trials Leukotriene receptor antagonists-2 trials Th2 cytokine inhibitor-1 trial	Theophylline-1 trial Nedocromil sodium-2 trials
	2. Chronic Bronchitis N = 8 trials, 731 subjects	Epinastine-1 trial Ipratropium bromide-1 trial Theophylline-1 trial Iodinised glycerol-2 trials	Low dose N-acetylcysteine-1 trial Budenoside-1 trial
	3. COPD N = 8 trials, 8013 subjects	Fenspiridine-1 trial Fluticasone-1 trial Formoterol-1 trial Neltenexine-3 trials Helicidine-1 trial Oxtriphylline-1 trial High dose N-acetylcysteine-1 trial Salbutamol/Iprapropium bromide-1 trial Iprapropium bromide-1 trial	Budenoside-1 trial Codeine-1 trial Nesosteine-1 trial Oxitropium bromide-1 trial
	4. Reflux disease N = 5 trials, 258 subjects	Lansoprazole-1 trial Omeprazole-2 trials	Esoprazole-1 trial Omeprazole-1 trial
	5. Idiopathic cough N = 2 trials	Morphine-1 trial Speech pathology training-1 trial	
	6. Other respiratory illnesses	Codeine-2 trials Benzonatate being equivalent to Codeine-1 trial Moguiesteine being equivalent to Dextromethophran-1 trial Neltenexine-2 trials Sinecod linctus (butamirate) with a similar effect to that of small dose of Codeine-1 trial	
Overview of findings from Cochrane systematic review in cancer [12]**	Lung cancer patients, N = 7 trials	Brachytherapy in addition to EBRT resulted in higher improvements in cough at doses of 15 Gy in 3 fractions; 14-16 Gy in 2 fractions or 10 Gy in a single fraction	
	Lung cancer patients, N = 1	Photodynamic therapy (PDT) showing similar results to laser therapy; its role as main treatment option questionable.	
	Pharmacological treatments, N = 9 (4 with mixed sample of patients with respiratory illnesses including cancer. Results extrapolated for cancer patients only)	Codeine 30 mg + Phenyltoloxamine 10 mg bd-1 trial Dihydrocodeine-1 trial Hydropropizine (= Levodropropizine)-1 trial Levodropropizine equivalent to Dihydrocodeine-1 trial A Morphine derivative equivalent to Codeine in capsules (unclear dose) Sodium Cromoglycate 40 mg (2 puffs) Butamirate linctus (overall no effect, but effective in cancer subsample)	
Case studies and reviews	Cancer patients, often with advanced disease	Morphine, Methadone, Pholcodine, Quaifenesin, Hydromorphone (due to their antitussive activity) [review] [15] Benzonatate for opioid-resistant cough [16] Nebulized Morphine [17] Nebulized Lidocaine [18] Hydrocodone (phase II trial) 10 mg/d in divided doses [37]	
Experimental studies or studies in non-cancer patients		GABA_B _agonists (such as Baclofen) [7] Dextromethorphan 10-20 mg/4-6 hrs better than Codeine 20 mg [13] Moguestine 100 mg tid equivalent to Codeine 15-30 mg (non-cancer patients) [14] Nebulised Lidocaine [19,20,38] Levocloperastine (novel antitussive) [21] Paroxetine (in concomitant pruritus and cough) [22] Amitryptiline, Gabapentin, Carbamazepine (in chronic cough) [23] Thalidomide [24]	

*Most of the therapeutic options here are not relevant to lung cancer-related cough, unless a relevant respiratory pathology is also present

**Most studies received a '0' Jadad score representing studies with very low methodological quality.

Research into cancer-related cough was even more disappointing, and our Cochrane systematic review [12] identified only 17 studies meeting the inclusion criteria. Almost half were studies investigating the effects of brachytherapy and nine trials assessed the effectiveness of a number of drugs (Table 1). All brachytherapy trials were of very low quality (Jadad score of '0') and often there was lack of clarity in the papers about key methodological processes. Nevertheless, these studies suggest that treatment with brachytherapy may be appropriate in selected populations of lung cancer patients. The pharmacological studies were mostly of low quality using small samples, half had mixed samples of non-cancer and cancer patients and several were over 30 years old.

We also assessed other trials that were not included in the above reviews, phase II trials and experimental case studies. A randomized double blind crossover trial of 16 patients with chronic stable cough was included, receiving Dextromethorphan or Codeine (20 mg) [13]. Results showed that Dextromethorphan reduced the intensity of cough more than Codeine did (p < 0.0008) and was considered the better antitussive by the majority of patients. In another randomized trial in 119 patients with various respiratory illnesses and cough, Moguisteine 100 mg tid was shown to be equally effective as Codeine [14]. Homsi et al [15] assessed the effects of Hydrocodone in 25 patients (20 completed the study) in a phase II trial. Results suggested that Hydrocodone at a starting dose of 10 mg/day in divided doses relieved cough in 70% of the sample, although dose titrations were necessary. Doona & Walsh [16] reported three cases who achieved symptomatic relief in opioid-resistant cough using Benzonatate, while Stein & Min [17] reported a single case of a patient with metastatic cancer who was benefited from the use of Nebulised Morphine for paroxysmal cough and dyspnoea. The positive effect of Nebulised Lidocaine was shown in a case study of a palliative care patient [18] as well as case studies in patients with respiratory diseases [19,20]. Aliprandi et al [21] reviewed trials using a novel antitussive, Levocloperastine, suggesting this has an improved efficacy and side effect profile compared to other antitussives. The potential role of Paroxetine, Baclofen, Amitryptiline, Gabapentin, Thalidomide and Carbamazepine have also been suggested through experimental work or in reviews [7,22-24].

There are, however, some issues with some propositions in the literature, particularly in relation to the effects of opioids. For example Homsi et al [15] mentioned a rank order of antitussive effects of opioids: The preferred drug being Methadone (linctus), then Hydromorphone, then Morphine, then Codeine, then Oxycodone, in that order, citing Eddy et al [25] as source document. However, the summary evidence following each opioid in this 1957 monograph does not compare antitussive effects against a standard nor against equi-analgesic doses, and so does not support this ranking. Also, Hydromorphone is said to be four times as potent an antitussive as morphine but this is based on a study that showed comparable effects in tuberculosis-related cough between 10 mg Morphine and 2.5 mg Hydromorphone. Based on equi-analgesic dose conversions, no opioid appears to be superior to another. Homsi et al [15] state that Oxycodone is less potent than Morphine as an antitussive (citing ref .25) but in fact the monograph [25] states that they are equally effective. Furthermore, there may be a strong placebo effect with many of the medications presented above (including weak opioids), and whereas a placebo effect is clinically useful, it needs to be considered in the interpretation of the data available.

## 4. Recommendations

### 4.1. Assessment of cough

A thorough history is fundamental to identify the cause of cough and should be taken for each patient with lung cancer in order to identify the causes of cough. This assessment should include the type of cough (productive/non-productive), trigger factors, whether cough is nocturnal or day-time cough, its effects on quality of life or any specific concerns patients may have (e.g. fear of choking during a cough). This assessment should include a careful medication review. Use of a cough scale should be useful in identifying not only the frequency of cough but also its severity and distress. A visual analogue scale is recommended, until validated scales specific for cancer patients are available, which also could be used to assess the effectiveness and responsiveness of initiated interventions. Many of the lung cancer patients have comorbidities with other respiratory diseases (e.g. chronic obstructive pulmonary disease-COPD) and cough may be the result of the underlying respiratory pathology rather than the cancer. The timing of the start of cough is important; any change in cough since the diagnosis with cancer or any new cough is likely to be related with the cancer whereas more chronic cough may be related to underlying respiratory comorbidity. Iatrogenic causes of cough should also be considered, such as radiotherapy and certain medication (e.g. 'lone cough' from Gefitinib, Trastuzumab, Methotrexate, Busulphan, Bleomycin, antihypertensive drugs or ACE inhibitors). Lung cancer patients may not need new investigations, as they may already have x-rays or CT scans that can provide adequate information regarding the cancer-related causes of cough. It may, however, be necessary to carry out such investigations if the causes of cancer-related cough are not apparent from a detailed history.

### 4.2. Treating reversible causes of cough/specific pathology

The therapeutic overall goal should first be to treat reversible causes or specific pathology. If the patient has an identified underlying pathology potentially causing cough, he/she should be treated as per available guidelines (ie. BTS guidelines). In cough due to airway diseases such as COPD or asthma, treatments directed at the underlying condition should be used. This could be achieved with the use of inhaled bronchodilators and corticosteroids; for example, salbutamol has little effect in non-asthmatic cough but may be useful when bronchoconstriction is present. The same principle applies to anticholinergics and theophylline. Anticholinergics (e.g. Hyoscine) may be particularly important in the end of life, where the aim is to suppress secretions and subsequent cough. Oral corticosteroids (e.g. Prednisolone 30 mg once daily for 14 days) may also provide rapid relief of cough due to airway inflammation either as a direct result of tumour involvement or associated asthmatic/eosinophilic inflammation. With cough thought to be originating from gastro-oesophageal reflux, PPIs and H2-receptor antagonists may be tried. However, increasing evidence suggests that non-acid reflux (pH 4-7) may be associated with chronic coughing in some individuals. Metoclopramide and Domperidone are frequently used to promote GI motility and they should be considered in selected patients where non-acid reflux is suspected. The BTS guidelines [7] recommend that patients with cough receiving an ACE inhibitor should discontinue it. Even in patients where ACE inhibitor treatment pre-dates coughing, their sensitizing effect on the cough reflex is likely to worsen symptoms. Also, the BTS guidelines recommend that in prominent upper airway pathology, a trial of a topical corticosteroid is appropriate. Productive cough may indicate bronchiectasis, sinusitis or a lower respiratory tract infection, and the use of antibiotics may be appropriate.

Systemic chemotherapy, were indicated, can improve symptoms in lung cancer, and there are a number of studies with varied chemotherapy regimens, where symptoms and quality of life were secondary outcomes, showing improvements in symptoms including cough [i.e. [26-30]]. Rapid palliation of cough (and other thoracic symptoms) can particularly be achieved in small cell lung cancer. Furthermore, a Cochrane review has provided evidence that external beam radiation therapy of one or two fractions produces significant improvements in thoracic symptoms [31]. Hence, systemic chemotherapy and/or radiotherapy are cornerstones for symptom management in lung cancer and the use of brachytherapy should also be considered as shown in our Cochrane review.

### 4.3. Symptomatic management

Symptomatic treatment should start with demulcents such as Glycerol-based ones (2 positive trials of moderate quality for Glycerol [32,33]) and Simple Linctus. A 2-week course of steroids could be considered in patients with extrinsic airway compression, although this may not be appropriate for all patients. Centrally-acting opioids should be the next step primarily with Codeine linctus. While the evidence for Codeine derives from COPD trials, opioids most likely act on the central nervous system and their action may be the same regardless of aetiology. Morphine and Methadone could also be considered in patients failing to respond with the weak opioids before introducing peripherally-acting agents such as Levodropropizine, Moguisteine or Levocloperastine where available (see review of peripherally-acting antitussives by Dicpinigaitis [34]). Low dose of sustained release Morphine (ie. 5 mg and sometime 10 mg twice daily) may produce adequate relief of cough, but unlike pain, it seems that higher doses do not necessarily improve effectiveness in relation to cough. Constipation can be a distressing side effect for patients, and it should be a clinical consideration when opioids are prescribed. Agents with local anaesthetic properties could also be considered, such as Nebulised Lidocaine and Benzonatate (where available). The suggested dose of the above agents is presented in Table 2. Failing all these, there are a number of more experimental options that could be considered (with minimal evidence and significant toxicity/side effects), such as Baclofen, Thalidomide, Gabapentin, Carbamazepine or Amitriptyline. Figure 1 presents the steps that are recommended to be followed in the management of cough in lung cancer. Due to the distressing nature of cough in lung cancer patients, high level of symptom burden and poor survival, methods used need to provide improvements in cough in a short period of time and long-term treatment approaches should be avoided.

**Table 2:** Recommended dosages for antitussives, demulcents and topical anaesthetics

Medication	Dosage
Simple linctus	5 ml tds/qds
Dextromethorphan	10-15 mg tds/qds
Codeine	30-60 mg qds
Pholcodine	10 ml tds
Morphine (oramorph)	5 mg (single dose trial of oramorph; if effective 5-10 mg slow release morphine bd)
Diamorphine	5-10 mg CSCI/24 hrs
Methadone linctus	Single dose 2 mg (2 mL of 1 mg/mL solution)
Dihydrocodeine*	10 mg tds
Hydrocodone	5 mg bd
Inhaled cromoglycate	10 mg qds
Levodropropizine*	75 mg tds
Moguisteine*	100-200 mg tds
Levocloperastine*	20 mg tds
Nebulised Lidocaine#	5 ml of 0.2% tds
Nebulised Bupivacaine#	5 ml of 0.25% tds
Benzonatate*	100-200 mg qds
Prednisolone	30 mg daily for 2 weeks

t.d.s.: 3 times daily; q.d.s.: 4 times daily; csci: subcutaneously; b.d.: twice daily.

*Not available in the UK and some other countries.

#Avoid food/drinks for at least 1 hr; first dose as inpatient in case of reflex bronchospasm.

Initial cough management should focus on peripherally acting and intermittent treatment (i.e demulcents); more resistant symptoms require the addition of (or replacement by) centrally acting and continuous treatment. It is noted, however, that peripheral mechanisms may be different in cancer-ulcerated mucosa in localized cancer may result in more or less sensitivity to peripheral agents such as lidocaine or steroids. Furthermore, many patients with advanced cancer will be already receiving strong opioids for pain management which may mean that centrally acting approaches are maximized and less well established treatments should be explored. Behavioural interventions, such as speech pathology training [35] and vocal hygiene may have an adjunctive role in the management of cough in lung cancer, but as many related techniques are based in cough suppression they may not be all appropriate for lung cancer patients. Examples of related techniques include pursed lip breathing/relaxed throat breathing/valsalva swallow/replacing cough with swallowing/distraction, although none of these techniques have been tested in cancer patients and their use is based on experience and limited research from the wider respiratory illness field. Inhalations of menthol may also be suggested, based on the premises that menthol can reduce cough reflex sensitivity (via the TRPM8 channel) [36], although no research with patients experiencing a respiratory illness could be identified.

A combination of cancer-specific treatments and symptomatic control approaches is likely to be necessary in all patients. The specific balance of these treatments should be determined by the individual patient's needs and a logical stepwise approach to seeking reversible causes and introducing treatments.

## Level of evidence

With the exception of chemoradiotherapy and some treatments for respiratory diseases, the recommendations in the cough management pyramid of Figure 1 are based on low level of evidence (level III-non analytic studies-and IV-expert opinion). The grade of recommendation is D or GPP (good practice). In terms of level of confidence in the available data, the higher the pyramid level in Figure 1 the lower the confidence level.

## Conclusion

Cough is a symptom that has received little attention in cancer supportive care research and it is frequently undertreated in practice as there is a dearth of good quality evidence on which clinicians can base their treatment decisions. These guidelines provide an attempt to rationalize the available evidence and offer a sensible and practical way of managing cough in lung cancer patients. There is an urgent need for more high quality research in the management of cough. Also, as traditional antitussives and other cough suppressants have variable effectiveness and significant side effects, more novel cough treatments need to be developed in the future based on improved understanding of pathophysiological causes of cough in patients with cancer.

## Competing interests

The authors declare that they have no competing interests.

## Authors' contributions

Conception of study, set up of Task Group and coordination: AM.

Literature review: AM, JAS, MIB.

Task Group participation: All authors.

Development of guidelines/recommendations: All authors.

Drafting paper: AM.

All authors read and approved the final manuscript.
